# *Aerococcus urinae* Spondylodiscitis: A Case Report and Review of the Literature

**DOI:** 10.1155/crdi/1569042

**Published:** 2025-08-28

**Authors:** Suranthaniy S. Sivalingam, Virginie Prendki, Nicolas Garin

**Affiliations:** ^1^Division of Internal Medicine, Riviera Chablais Hospital, Rennaz, Switzerland; ^2^Division of Internal Medicine for the Aged, Geneva University Hospitals, Geneva, Switzerland

**Keywords:** *Aerococcus urinae*, bone infection, spondylodiscitis

## Abstract

**Background:** *Aerococcus urinae*, a rare human pathogen, mainly causes urinary tract infection, endocarditis, and bacteremia. However, it is rarely the cause of other types of infection such as spondylodiscitis. Invasive *A. urinae* infection chiefly occurs in older men with underlying urinary tract disorders. The real incidence may be underestimated, as *Aerococci* grow in a CO2-containing atmosphere, and urine cultures are usually not incubated in this environment. There have been eight case reports of spondylodiscitis due to *A. urinae.*

**Material and Methods:** We report a 9^th^ case occurring in an 80-year-old Caucasian man with lower back pain. The patient had predisposing conditions (diabetes and a history of urological surgery). Spinal MRI showed signs of spondylodiscitis. Two computed tomography–guided biopsies targeting the intervertebral disc and psoas muscle were not diagnostic. One of 10 blood culture bottles grew *A. urinae*.

**Results:** After worsening of an L2 fracture, and unsuccessful percutaneous sampling procedures, the patient underwent surgical stabilization of T12 to L4 with multiple biopsies. Biopsy cultures grew *Aerococcus urinae*. Amoxicillin was administered intravenously for 14 days, followed by oral levofloxacin for 3 months.

**Conclusion:** *A. urinae* should be considered in spondylodiscitis with negative cultures, particularly in older men with diabetes and urological conditions.

## 1. Introduction

*Aerococcus urinae* is a rare human pathogen that primarily causes urinary tract infection, endocarditis, and bacteremia. However, it is infrequently responsible for other types of infection such as spondylodiscitis. Invasive *A. urinae* infection chiefly occurs in older men with underlying urinary tract disorders. The real incidence may be underestimated, as *Aerococci* grow in a CO_2_-containing atmosphere, and urine cultures are usually not incubated in this environment. Although bacteremia and endocarditis are the predominant clinical manifestations, *A. urinae* has also been implicated in other severe infections, including spondylodiscitis. To date, only eight cases of *A. urinae* spondylodiscitis have been reported in the literature. Here, we describe a ninth case, emphasizing the clinical presentation, diagnostic challenges, and therapeutic management of this rare condition in the context of an emerging pathogen with evolving antibiotic resistance.

## 2. Case Description

**Figure 1:**
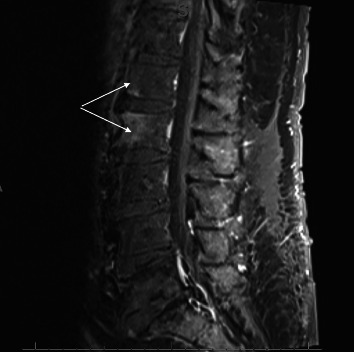
First lumbar MRI (16.12) with white arrow showing signs of L1-L2 spondylitis.

An 80-year-old Caucasian man was admitted to the hospital with lower back pain for four days. There was no history of trauma, fever, or weight loss. His past medical history was notable for Type 2 diabetes, urethrotomy and meatotomy, orchiepididymitis, and *Escherichia coli* and *Actinotignum schaalii* bacteremia. He did not have an indwelling urinary catheter. Palpation of the spinous processes from L1 to L5 was painful. C-reactive protein was elevated (89 mg/L), but the blood count was normal. A computed tomography (CT) scan showed degenerative changes, causing nerve root impingement, and a circumferential disco-osteophytic bulge in L5-S1. Magnetic resonance imaging (MRI) revealed signs of spondylitis of L1-L2 bodies without associated discitis, along with inflammation of the paravertebral soft tissues (Figure 1); see also Table 1 for the clinical timeline.

### 2.1. Methods and Procedures

**Figure 3:**
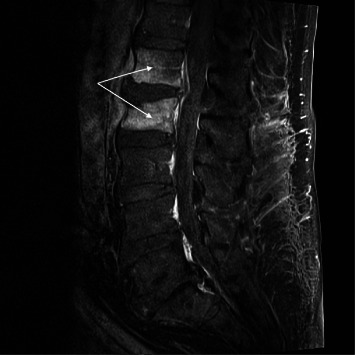
Second MRI sagittal (30.12) T1 fat sat injected. White arrow showing contrast-enhancing inflammatory changes in L1 and L2 vertebrae with preserved intervertebral disc.

A percutaneous CT scan–guided intervertebral disc sample was obtained under intravenous sedation. Anaerobic and aerobic cultures were sterile, and the eubacteria polymerase chain reaction (PCR) targeting 16S rRNA was negative. A second MRI three weeks after symptoms onset showed progressive inflammatory changes, L1-L2 spondylodiscitis, and a compression fracture of the upper end plate of L2 (Figure 3). Paravertebral soft tissue infiltration had now extended to the psoas and paravertebral muscles. A second CT-guided biopsy targeting the psoas muscle was performed under local anesthesia. The cultures were again sterile; *Mycoplasma* and eubacteria PCR were negative.

The patient presented with intermittent fever, and multiple blood cultures were obtained. One of the ten bottles was positive for *Aerococcus urinae*, while urine cultures were sterile despite the presence of pyuria and hematuria on urinalysis. Due to increasing pain, worsening of the L2 fracture, and unsuccessful percutaneous procedures, the patient underwent open surgery with stabilization from T12 to L4 by pedicle screws, four weeks after admission. Multiple biopsies were obtained. Biopsy cultures grew *A. urinae*. These clinical events are summarized in Table 1. The minimal inhibitory concentrations were as follows: amoxicillin 0.023 μg/mL, levofloxacin 0.25 μg/mL, ciprofloxacin 0.094 μg/mL, and clindamycin 0.125 μg/mL.

Amoxicillin 2 g tid was administered intravenously for 14 days, followed by oral levofloxacin 500 mg bid for 3 months. Levofloxacin was chosen for the oral therapy due to the high bone concentration achievable with quinolones.

**Figure 4:**
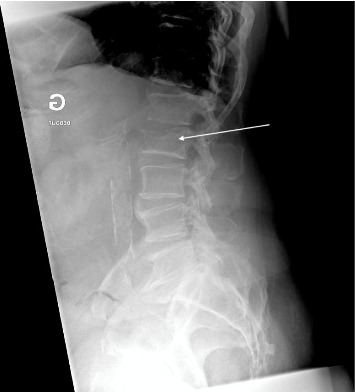
X-ray (03.01) white arrow showing loss of height of L2 with signs of bone destruction.

At 4 months, the outcome was partially favorable, with resolution of inflammation but persisting back pain and destruction of L2 and the inferior part of L1 (Figure 4).

## 3. Discussion and Conclusion

*A. urinae* was identified as a new species in 1992. It is a Gram-positive coccus and an alpha-hemolytic facultative anaerobic bacterium that grows in clusters and is catalase-negative [1].

*Aerococci* are found in 0.2%–0.8% of urine cultures. The predominant species is *A. urinae* (55%–66% of isolates) [1]. In a Swiss study from 2012, *A. urinae* was the 6^th^ most common pathogen, being present in 4% of urinary tract infections [2]. *Aeroccoci* bacteriuria is more frequent in the elderly [1]. Invasive *A. urinae* infection mainly occurs in older men with underlying urinary tract conditions [3]. A recent study found an incidence of *A. urinae* bacteremia of 9.2 cases per 1,000,000 inhabitants per year, with a 22% mortality rate [4].

Apart from urinary tract infections, *A. urinae* mainly causes bacteremia and endocarditis [1, 5]. Less common infections are peritonitis, soft tissue infections of the genital area, postpartum infections, odontogenic infections, joint infections, postoperative infections, and vertebral osteomyelitis [1, 4].

The real incidence may be underestimated, as *Aeroco*cci grow in a CO_2_-containing atmosphere, and urine cultures are usually not incubated in this environment [1]. The reference standard for identification requires sequencing of the 16S rRNA [3]. Use of matrix-assisted laser desorption ionization time-of-flight mass spectrometry (MALDI-TOF MS) has led to increased identification of *Aerococcal* infection, and its widespread use should enable timely identification of unusual or unexpected pathogens [6]. Indeed, MALDI-TOF MS is routinely used at our institution for rapid identification of pathogens in blood cultures. The Gram-positive coccus present in the blood culture of our patient was identified as *A. urinae* less than 24 h after growth was detected.

Treatment recommendations are based on series, case reports, and in vitro findings. *Aerococci* are generally susceptible to beta-lactam antibiotics and resistant to sulfamethoxazole [1]. *A. urinae* has a very low minimum inhibitory concentration for penicillin, cephalosporin, and carbapenem [3]. However, a recent study found increasing resistance with some isolates nonsusceptible to penicillin, cephalosporin, and fluoroquinolones [7].

As for spondylodiscitis, it predominantly affects older men with chronic comorbidities [8]. Risk factors are diabetes, chronic kidney disease, excessive alcohol consumption, intravenous drug use, recent infection of the genitourinary system, and previous spinal surgery [9, 10]. *Staphylococcus aureus* is the most common pathogen (43%–59%). *Escherichia coli, Proteus* sp, *Klebsiella* sp, and *Pseudomonas aeruginosa* are other possible pathogens [9]. The infection is usually hematogenous and rarely occurs by direct inoculation of the spine. The pathogen is present in blood cultures in 25%–59%. According to guidelines from the Infectious Diseases Society of America, the diagnostic evaluation requires obtaining two sets of blood culture, an MRI (which has a sensitivity of 97% and a specificity of 93%), and a CT scan–guided aspiration biopsy if blood cultures are sterile [8]. If no pathogen is identified in the first biopsy, unusual pathogens should be considered (anaerobes, fungi, *Brucella*, and *Mycobacteria*), and repeating the procedure is frequently preferred over proceeding directly with an open biopsy. Though it is a less invasive approach, it also has drawbacks. First, the ability to lie still during the procedure can be compromised in elderly patients with severe back pain, which may prevent the acquisition of good-quality samples. Furthermore, the sensitivity of CT scan–guided biopsy is only 30%–74%, dropping as low as 48% in a recent meta-analysis [11]. CT scan–guided biopsies have a significantly lower yield compared to open surgical biopsies [11]. Considering the prolonged duration of treatment (between 4 and 12 weeks), obtaining an etiologic pathogen should be a priority, and empiric treatment should be withheld in stable patients without neurological compromise [8].

We searched PubMed and Google Scholar with the keywords “Spondylodiscitis, Spine infection, bone infection” together with *“Aerococcus*.” We found only 8 cases of *A. urinae* spondylodiscitis (Table 2). Seven patients were men, and six were more than 65 years old. Three patients had diabetes, and four had an underlying urological condition. All bone biopsies were positive for *A. urinae*. Five of seven blood cultures and only one of seven urine cultures were positive. Seven cases were initially treated with a beta-lactam (amoxicillin, penicillin, and ampicillin) as the only antibiotic (3/8) or associated with clindamycin (2/8) or gentamicin (2/8). The treatment was switched to amoxicillin per os (3/8), or a fluoroquinolone (3/8) with (2/8) or without (1/8) clindamycin. The total duration of the antibiotic treatment was at least 4 weeks, with an average of 12 weeks. All cases had a successful outcome. An additional table displays more details of the 9 cases (see additional file 1). Our case had similar demographic and clinical features. However, our patient did not have a successful outcome. This could be related to the prolonged diagnostic process before identification of the pathogen (28 days). However, the median time to diagnosis was 30 days in a recent review of 207 patients, in line with our case [20]. Whether a more aggressive approach, with escalation to open surgery after the negative result of the first CT scan–guided aspiration, would have led to a better outcome remains unresolved. Recommendations to escalate to open biopsy (progressive neurologic deficits, deformity, or failure to control a bloodstream infection) were not met before repeated imaging showed collapse of L2 [8].

In conclusion, we present a case of *Aerococcus urinae* spondylodiscitis identified in a surgical biopsy obtained four weeks after admission and in one of ten blood cultures. Urine culture and two CT scan–guided spinal samples were negative, highlighting the difficulty of isolating this pathogen from urine and the limited sensitivity of CT scan–guided biopsy. *Aerococcus urinae* should be considered in older men with urological conditions and spondylodiscitis of undetermined cause.

## Figures and Tables

**Figure 2:**
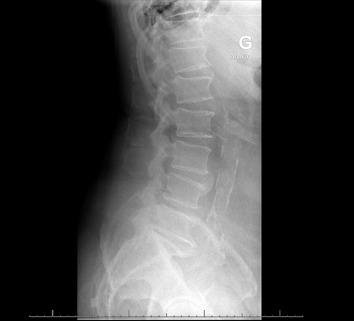
Lumbar X-ray (11.12): no signs of bone destruction.

**Table 1:** Clinical timeline of patient's investigations and intervention.

Date	Event
11.12	Admission with RX (Figure 2)
12.12	First CT scan
16.12	First MRI (Figure 1)
23.12	First CT scan–guided biopsy
30.12	Second MRI (Figure 3)
03.01	Second biopsy X-ray showing L2 collapse (Figure 4)
05.01	Positive blood culture for Gram-positive cocci
06.01	Identification of *Aerococcus urinae* in blood culture
09.01	Surgical intervention

**Table 2:** Characteristics of 9 published cases of *Aerococcus urinae* spondylodiscitis, their risk factors, microbiological findings, antibiotics regimens, and outcomes.

Study	Sex, age	Risk factors	Blood culture	Urine culture	Bone culture	Antibiotic regimen	Outcome
Astudillo et al. [12]	F, 76 y	Diabetes, renal failure	Pos	Neg	None	AMX IV plus clindamycin IV 1 month, then AMX PO during 5 months	Favorable after 2 months
Tekin et al. [13]	M, 68 y	Diabetes, prostatic hyperplasia	Pos	Neg	None	Penicillin G IV plus gentamycin IV during 4 weeks then oral antibiotic (no data)	Favorable after 3 weeks
Jerome et al. [14]	M, 37 y	Obesity, urethral strictures	None	None	Pos	Cefazolin for 6 weeks	Favorable
Torres-Mortos et al. [15]	M, 79 y	Prostatic hyperplasia	Neg	Pos	Pos	AMX IV during 3 weeks then AMX per os for 4 months	Favorable after 3 weeks
Degroote et al. [16]	M, 32 y	Paraplegia	Neg	Neg	Pos	Penicillin G IV during 2 weeks then ciprofloxacin plus clindamycin PO during 8 weeks	Favorable after 3 weeks
Rougier et al. [17]	M, 81 y	Sjogren syndrome	Pos	Pos	Pos	AMX IV plus clindamycin IV then levofloxacin plus clindamycin PO for 6 weeks total	Favorable
Lyagoubi et al. [18]	M, 77 y	Prostatic hyperplasia	Pos	Pos	Pos	AMX IV for 2 weeks then AMX PO for 6 weeks	Favorable after 2 weeks
Butcher et al. [19]	M, 74 y	Obesity, diabetes, and chronic kidney disease	Pos	Pos	None	AMX PO for 10 days then levofloxacin for 6 weeks	Favorable after 2 months
Our case	M, 80 y	Diabetes, urethrotomy, and meatotomy	Pos	Neg	Pos	AMX iv for 14 days then levofloxacin for 3 months	Persisting back pain and destruction of L2 and partly of L1

Abbreviations: AMX, amoxicillin; IV, intravenously; PO, per orale.

## Data Availability

All data generated and/or analyzed during the current study are available from the corresponding author on reasonable request.

## References

[1] Rasmussen M. (2016). Aerococcus: An Increasingly Acknowledged Human Pathogen. *Clinical Microbiology and Infection*.

[2] Guilarte Y. N., Tinguely R., Lupo A., Endimiani A. (2014). Prevalence and Characteristics of Fluoroquinolone-Resistant *Aerococcus urinae* Isolates Detected in Switzerland. *International Journal of Antimicrobial Agents*.

[3] Rasmussen M. (2013). Aerococci and Aerococcal Infections. *Journal of Infection*.

[4] Sihvonen R., Turunen M., Lehtola L. (2022). Clinical and Microbiological Characterization of *Aerococcus urinae* Bacteraemias at Helsinki Metropolitan Area, Finland. *European Journal of Clinical Microbiology and Infectious Diseases*.

[5] Tiong C. W., Bartolo C., Walton A., Athan E. (2022). *Aerococcus urinae*, a Rare Cause of Aortic Root Abscess: A Case Report. *Journal of Medical Case Reports*.

[6] Senneby E., Nilson B., Petersson A. C., Rasmussen M. (2013). Matrix-Assisted Laser Desorption Ionization-Time of Flight Mass Spectrometry Is a Sensitive and Specific Method for Identification of Aerococci. *Journal of Clinical Microbiology*.

[7] Lupo A., Guilarte Y. N., Droz S., Hirzel C., Furrer H., Endimiani A. (2014). In Vitro Activity of Clinically Implemented Beta-Lactams Against Aerococcus Urinae: Presence of Non-Susceptible Isolates in Switzerland. *New Microbiologica*.

[8] Berbari E. F., Kanj S. S., Kowalski T. J. (2015). 2015 Infectious Diseases Society of America (IDSA) Clinical Practice Guidelines for the Diagnosis and Treatment of Native Vertebral Osteomyelitis in Adultsa. *Clinical Infectious Diseases*.

[9] Baryeh K., Anazor F., Iyer S., Rajagopal T. (2022). Spondylodiscitis in Adults: Diagnosis and Management. *British Journal of Hospital Medicine*.

[10] Kourbeti I. S., Tsiodras S., Boumpas D. T. (2008). Spinal Infections: Evolving Concepts. *Current Opinion in Rheumatology*.

[11] McNamara A. L., Dickerson E. C., Gomez-Hassan D. M., Cinti S. K., Srinivasan A. (2017). Yield of Image-Guided Needle Biopsy for Infectious Discitis: A Systematic Review and Meta-Analysis. *American Journal of Neuroradiology*.

[12] Astudillo L., Sailler L., Porte L., Lefevre J. C., Massip P., Arlet-Suau E. (2003). Spondylodiscitis due to Aerococcus Urinae: A First Report. *Scandinavian Journal of Infectious Diseases*.

[13] Tekin A., Tekin G., Turunc T., Demiroğlu Z., Kızılkılıç O. (2007). Infective Endocarditis and Spondylodiscitis in a Patient due to Aerococcus Urinae: First Report. *International Journal of Cardiology*.

[14] Jerome M., Slim J., Sison R., Marton R. (2015). A Case of *Aerococcus urinae* Vertebral Osteomyelitis. *Journal of Global Infectious Diseases*.

[15] Torres-Martos E., Perez-Cortes S., Sanchez-Calvo J. M., Lopez-Prieto M. D. (2017). Spondylodiscitis due to *Aerococcus urinae* Infection in an Elderly Immunocompetent Patient. *Enfermedades Infecciosas Y Microbiología Clínica*.

[16] Degroote E., Yildiz H., Lecouvet F., Verroken A., Belkhir L. (2018). *Aerococcus urinae*: An Underestimated Cause of Spine Infection? Case Report and Review of the Literature. *Acta Clinica Belgica*.

[17] Rougier E., Braud A., Argemi X. (2018). Spondylodiscitis due to *Aerococcus urinae* and Literature Review. *Infection*.

[18] Lyagoubi A., Souffi C., Baroiller V., Vallee E. (2020). *Aerococcus urinae* Spondylodiscitis: An Increasingly Described Localization. *EJIFCC*.

[19] Butcher B., Holman E., Johnson J. R., Boothby A. (2022). Oral Therapy for *Aerococcus urinae* Bacteremia and Thoracic Spondylodiscitis of Presumed Urinary Origin. *Federal Practitioner: For the Health Care Professionals of the VA, DoD, and PHS*.

[20] Pola E., Taccari F., Autore G. (2018). Multidisciplinary Management of Pyogenic Spondylodiscitis: Epidemiological and Clinical Features, Prognostic Factors and Long-Term Outcomes in 207 Patients. *European Spine Journal*.

